# Human Milk and Donkey Milk, Compared to Cow Milk, Reduce Inflammatory Mediators and Modulate Glucose and Lipid Metabolism, Acting on Mitochondrial Function and Oleylethanolamide Levels in Rat Skeletal Muscle

**DOI:** 10.3389/fphys.2018.00032

**Published:** 2018-01-30

**Authors:** Giovanna Trinchese, Gina Cavaliere, Chiara De Filippo, Serena Aceto, Marina Prisco, Jong Tai Chun, Eduardo Penna, Rossella Negri, Laura Muredda, Andrea Demurtas, Sebastiano Banni, Roberto Berni-Canani, Giuseppina Mattace Raso, Antonio Calignano, Rosaria Meli, Luigi Greco, Marianna Crispino, Maria P. Mollica

**Affiliations:** ^1^Department of Biology, University of Naples Federico II, Naples, Italy; ^2^Biology and Evolution of Marine Organisms, Stazione Zoologica Anton Dohrn, Naples, Italy; ^3^European Laboratory for Food Induced Diseases, Department of Translational Medical Sciences, University of Naples Federico II, Naples, Italy; ^4^Dipartimento di Scienze Biomediche, Università degli Studi di Cagliari, Cagliari, Italy; ^5^Department of Pharmacy, University of Naples Federico II, Naples, Italy

**Keywords:** human milk, donkey milk, mitochondrial functions, mitochondrial dynamics, oxidative stress, inflammation

## Abstract

**Scope:** Milk from various species differs in nutrient composition. In particular, human milk (HM) and donkey milk (DM) are characterized by a relative high level of triacylglycerol enriched in palmitic acid in sn-2 position. These dietary fats seem to exert beneficial nutritional properties through N-acylethanolamine tissue modulation. The aim of this study is to compare the effects of cow milk (CM), DM, and HM on inflammation and glucose and lipid metabolism, focusing on mitochondrial function, efficiency, and dynamics in skeletal muscle, which is the major determinant of resting metabolic rate. Moreover, we also evaluated the levels of endocannabinoids and N-acylethanolamines in liver and skeletal muscle, since tissue fatty acid profiles can be modulated by nutrient intervention.

**Procedures:** To this aim, rats were fed with CM, DM, or HM for 4 weeks. Then, glucose tolerance and insulin resistance were analyzed. Pro-inflammatory and anti-inflammatory cytokines were evaluated in serum and skeletal muscle. Skeletal muscle was also processed to estimate mitochondrial function, efficiency, and dynamics, oxidative stress, and antioxidant/detoxifying enzyme activities. Fatty acid profiles, endocannabinoids, and N-acylethanolamine congeners were determined in liver and skeletal muscle tissue.

**Results:** We demonstrated that DM or HM administration reducing inflammation status, improves glucose disposal and insulin resistance and reduces lipid accumulation in skeletal muscle. Moreover, HM or DM administration increases redox status, and mitochondrial uncoupling, affecting mitochondrial dynamics in the skeletal muscle. Interestingly, HM and DM supplementation increase liver and muscle levels of the N-oleoylethanolamine (OEA), a key regulator of lipid metabolism and inflammation.

**Conclusions:** HM and DM have a healthy nutritional effect, acting on inflammatory factors and glucose and lipid metabolism. This beneficial effect is associated to a modulation of mitochondrial function, efficiency, and dynamics and to an increase of OEA levels in skeletal muscle.

## Introduction

Human milk (HM), the natural food of infants, provides an adequate supply of all nutrients necessary to support postnatal growth and development. In addition, it provides immunomodulant components and plays a key role in preventing inflammation and metabolic diseases throughout life (Agostoni et al., 2013; Cacho and Lawrence, 2017). Interestingly, it has been demonstrated that breastfed individuals have lower rate of obesity and type 2 diabetes than those fed with infant formula (Owen et al., 2006). Such effects have been attributed to appetite regulation and reduced weight gain in breast-fed children and/or to the presence of unique nutrients or bioactive constituents in HM. In fact, metabolism-regulating hormones, such as leptin, adiponectin, resistin and ghrelin, detected in HM (Lönnerdal, 2013), may be involved in the regulation of growth in infancy and might influence the programming of energy balance with long-term consequences on health (Agostoni et al., 2013). In addition, the peculiar high concentration in HM of palmitic acid in the sn-2 position of triacylglycerol backbone seems to play a crucial role in the regulation of energy and lipid metabolism (Innis, 2016). Interestingly, also donkey milk (DM) is characterized by a high concentration of palmitic acid in sn-2 position of triacylglycerol, and is recognized as the best potential substitute for HM due to its remarkable nutritional value coupled with good palatability and reduced allergenicity (Tafaro et al., 2007; Fiocchi et al., 2010). Recently, we have demonstrated that palmitic acid in sn-2 position of triacylglycerol is able to modulate N-acylethanolamine levels in rat tissues, which may possibly explain its effects on lipid and energy metabolism (Carta et al., 2015). Among N-acylethanolamine, palmitoylethanolamide (PEA) and oleylethanolamide (OEA), are PPARα endogenous agonists. PPARs are a family of nuclear receptors that regulate fatty acid metabolism in different tissues. In particular, PPARα activation increases peroxisomal and mitochondrial oxidation and leads to anti-inflammatory and antioxidant effects (Fu et al., 2005; Lo Verme et al., 2005; Contreras et al., 2013).

Recently, we have demonstrated that HM or DM administration improves liver inflammatory state, enhancing hepatic mitochondrial functions and uncoupling (Trinchese et al., 2015). Mitochondrial uncoupling, dissipating the proton gradient across the inner membrane, creates a futile cycle of glucose and fatty acid oxidation without generating ATP (Nedergaard et al., 2005; Tseng et al., 2010), and thereby increases lipid oxidation and reduces intracellular lipid content (Harper et al., 2008). Promoting this inefficient metabolism that generates heat instead of ATP, mitochondrial uncoupling can serve as a potential treatment for obesity. Therefore, drugs, nutrients, or natural molecules modulating the mitochondrial function and efficiency can be considered as potential treatment for obesity and insulin resistance promoting an inefficient metabolism (Li et al., 2000). To ensure a healthy mitochondrial population, cells are equipped with various quality control systems, that regulate mitochondrial shape, function, and mass. These regulatory systems include mitochondrial biogenesis and dynamics (Liesa and Shirihai, 2013). In particular, mitochondrial dynamic is a tightly regulated process controlled by fusion and fission events (Detmer and Chan, 2007). During mitochondrial fusion, optic atrophy (OPA)1 is responsible for mitochondrial inner membrane fusion, and two other proteins mitofusins 1 and 2 (Mfn1 and Mfn2) mediate outer membrane fusion. On the other hand, dynamin-related protein (DRP)1 and fission protein (Fis)1 are involved in mitochondrial fission (Detmer and Chan, 2007). Usually, a shift toward fusion optimizes mitochondrial function and plays a beneficial role in the maintenance of long-term bioenergetic capacity. In contrast, a shift toward fission results in numerous mitochondrial fragments contributing to elimination of irreversibly damaged mitochondria through autophagy. Not surprisingly, alteration of mitochondrial dynamics has profound impact on several pathological conditions including metabolic diseases such as obesity and type 2 diabetes (Sebastián et al., 2012; Schneeberger et al., 2013; Zorzano et al., 2015). Skeletal muscle is one of the major targets of insulin accounting for high percentage of the hormone-dependent glucose uptake. Indeed, metabolic impairment in skeletal muscle strongly affects glucose disposal in the whole-body. Moreover, skeletal muscle is a chief determinant of resting metabolic rate, whose reduction is associated with weight gain (Ravussin et al., 1988). Recent data have demonstrated that the insulin-resistant condition in skeletal muscle is characterized by alterations of mitochondrial function and efficiency (Cavaliere et al., 2016).

Here, we examined the effects of supplementation of cow milk (CM), HM, and DM on systemic and skeletal muscle inflammation, insulin sensitivity, endogenous lipids, and endocannabinoids levels in skeletal muscle and liver, and mitochondrial functions in skeletal muscle.

## Materials and methods

### Animals and chemicals

All chemicals were purchased from Sigma–Aldrich (St. Louis, MO, USA), unless specified otherwise. Male Wistar rats (60 days old; 345 ± 7 g; Charles River, Calco, Lecco, Italy) were caged in a temperature-controlled room and exposed to a daily light–dark cycle (12/12 h) with free access to chow diet and drinking water. The rats were divided into four experimental groups (*n* = 7), three of them were supplemented with equicaloric intake (82 kJ) of raw CM, DM, or HM drinking 21, 48, or 22 ml/day, respectively. The animals were treated for 4 weeks. The last group did not receive milk supplement and was used as control. Despite the different volumes used, the energy density provided by the different milk supplements was kept virtually the same (Table 1). DM from the Ragusana breed and CM were obtained from “Azienda Agricola Insalata” (Oliveto Citra, SA, Italy). Human milk was kindly provided by the milk bank “Banca del Latte Materno” Ospedale Colle dell'Ara—Chieti-Italy. At the end of the treatments, the animals were anesthetized by intra-peritoneal injection of chloral hydrate (40 mg/100 g body weight), and blood was taken from the inferior cava. Skeletal muscle was removed from the hind leg for analysis, and the samples not immediately used for mitochondrial preparation were stored at −80°C. All experiments were conducted in compliance with national guidelines for the care and use of research animals (D.L. 116/92, implementation of EEC directive 609/86).

**Table 1:** Energy intake and body weight gain.

	**Control**	**CM**	**DM**	**HM**
Initial body weight (g)	322 ± 3.0^a^	325 ± 4^a^	322 ± 3.9^a^	320 ± 4.7^a^
Final body weight (g)	470 ± 2.5^a^	505 ± 4.8^b^	464 ± 4^a^	460 ± 4.4^a^
Body weight gain (g)	148 ± 3.1^a^	180 ± 3.1^b^	142 ± 1.3^a^	140 ± 0.9^a^
Standard diet intake (kJ)	12, 448 ± 166^a^	12, 133 ± 203^a^	12, 140 ± 253^a^	12, 153 ± 187^a^
Milk intake (kJ)		2, 007 ± 15^a^	2, 000 ± 14^a^	2, 002 ± 21^a^
Total intake (kJ)	12, 448 ± 166^a^	14, 129 ± 204^b^	14, 140 ± 226^b^	14, 155 ± 212^b^

*Values represent means ± SEM from n = 7 animals/group. Labeled means without a common letter differ (P < 0.05)*.

### Serum and tissue parameters

Plasma concentrations of triglycerides and cholesterol were measured by colorimetric enzyme reaction by use of commercial kits (SGM Italia, Italy and Randox Laboratories ltd., United Kingdom). Instead, specific ELISA kits were used to measure the serum and tissue levels of IL-1α, IL-10 (Thermo Scientific, Rockford, IL, USA), TNF-α, and monocyte chemoattractant protein-1 (MCP-1) (Biovendor R&D, Brno, Czech Republic), adiponectin and leptin (B-Bridge International Mountain View, CA).

### Oral glucose tolerance test and insulin tolerance test

For oral glucose tolerance test, rats were allowed to fast overnight and then orally dosed with glucose (3 g/kg body weight) dissolved in water. For insulin tolerance test, fasting rats (5 h) were intraperitoneally injected with insulin (homolog-rapid-acting, 10 unit/kg body weight in sterile saline; Novartis, Basel, Switzerland). Blood was collected by direct flow from a small tail cut, before and after the treatments at certain intervals, and the glucose levels were determined by glucose monitor calibrated for rats (BRIO, Ascensia, NY), and the insulin levels by ELISA (Mercodia rat insulin; Mercodia, Uppsala, Sweden). Basal fasting values of serum glucose and insulin were used to calculate Homoeostatic Model Assessment (HOMA) index as [Glucose (mg/dL) ^*^ Insulin (mU/L)]/405 (Cacho et al., 2008).

### Mitochondrial parameters and basal and inducible proton leak

Mitochondrial isolation, oxygen consumption, and proton leakage measurements were performed as previously reported (Cavaliere et al., 2016). Oxygen consumption (polarographically measured using a Clark-type electrode) was measured in the presence of substrates and ADP (state 3) or with substrates alone (state 4), and respiratory control ratio (RCR) was calculated. Mitochondrial proton leakage was assessed by a titration of the steady-state respiration rate as a function of the mitochondrial membrane potential in skeletal muscle mitochondria. Carnitine palmitoyl-transferase (CPT) system, aconitase, and superoxide dismutase (SOD) specific activity were measured spectrophotometrically as previously reported (Flohe and Otting, 1984; Alexson and Nedergaard, 1988; Hausladen and Fridovich, 1996). Rate of mitochondrial H_2_O_2_ release was assayed by following the linear increase in fluorescence (ex 312 nm and em 420 nm) due to the oxidation of homovanillic acid in the presence of horseradish peroxidase (Barja, 1998).

### Lipid content, redox status, and Nrf2-activated enzyme activities in skeletal muscles

Total lipid content in the skeletal muscle was estimated by using the Folch method. Briefly, the skeletal muscle was weighed, chopped into small pieces, thoroughly mixed, and finally homogenized with water (volumes equal to twice the skeletal muscle weight) in a Polytron homogenizer. On appropriate aliquots of the homogenate, lipid content was determined gravimetrically after extraction in chloroform–methanol and evaporation to constant weight by a rotating evaporator (Folch et al., 1957).

Reduced (GSH) and oxidized (GSSG) glutathione concentrations in skeletal muscle were measured with the dithionitrobenzoic acid (DTNB)-GSSG reductase recycling assay (Bergamo et al., 2007), and the GSH/GSSG ratio was used as an oxidative stress marker. To investigate the possible involvement of NF-E2-related factor 2 (Nrf2) in the diet-induced stress, cytoplasmic extracts were prepared from rat skeletal muscle. The enzymatic activities of glutathione S-transferases (GSTs) and quinone-oxidoreductase (NQO1) were evaluated spectrophotometrically in cytoplasmic extracts (Benson et al., 1980; Habig and Jakoby, 1981; Levine et al., 1990).

### Lipid analysis

Aliquots of total lipid extract from tissues were mildly saponified as previously described (Banni et al., 1996) in order to obtain free fatty acids for HPLC analysis. Separation of unsaturated fatty acids was carried out with an Agilent 1100 HPLC system (Agilent, Palo Alto, Calif., USA) equipped with a diode array detector as previously reported. Spectra (195–315 nm) of the eluate were obtained every 1.28 s and were electronically stored. These spectra were acquired to confirm the identified HPLC peaks (Melis et al., 2001). Quantification of anandamide (AEA), 2-arachidonoyl glycerol (2-AG), PEA, and OEA was conducted by liquid chromatography-atmospheric pressure chemical ionization-mass spectrometry (LC-APCI-MS) using as internal standards their deuterated homologs as previously described (Piscitelli et al., 2011).

### Real time PCR analysis

Total RNA was extracted from skeletal muscle using the TRIzol reagent (Ambion). After DNase treatment, RNA was quantified and reverse-transcribed (1 μg) using the Advantage RT-PCR kit (Clontech). For the evaluation of mitochondrial fission and fusion gene transcription, we used murine primers, whose sequences are shown in Table 2. qPCR was performed as previously described (Trinchese et al., 2015).

**Table 2:** The sequences (5′-3′) of the primers used in Real Time PCR.

**Gene**	**Forward**	**Reverse**
*Mfn1*	TCTCCAAGCCCAACATCTTCA	ACTCCGGCTCCGAAGCA
*Mfn2*	TCAATGGCATCTTTGAGCAG	TCCAGGACCTGCTCTTCTGT
*Drp1*	ATCCAGCTGCCTCAGATTGT	GTGACCACACCAGTCCCTCT
*Opa1*	CAGAAGACCTCGCCAATTTC	TGTCACTTTCGGATCCATGA
*Fis1*	GAAGTATGTGCGGGGACTGT	CCATGCCTACCAGTCCATCT
*βactin*	ATTGCTGACAGGATGCAGAA	TAGAGCCACCAATCCACACAG

### Western blot analysis

Tissues were homogenized in lysis buffer (150 mM NaCl, 1 mM EDTA, 1 mM EGTA, 1% Triton X-100, Tris-HCl 20 mM, pH 7.5) containing protease and phosphatase inhibitors. Proteins (15–30 μg/lane) were separated on 10–12% SDS-polyacrilamide gel and transferred to PVDF membranes (GE Healthcare). Blocking (5% non-fat milk, 30 min) and immunoreaction (o/n) were performed in TBST buffer (0.1% Tween, 150mM NaCl, 10mM Tris-HCl, pH 7.5) at room temperature. The primary antibodies were anti-Mitofusin1, anti-Mitofusin2, anti-DRP1 (all Santa Cruz), and anti-β-actin (BD Biosciences). Signals were visualized by horseradish peroxidase-linked secondary antibodies in enzyme-linked chemiluminescence (ECL, Millipore). Relative protein levels were normalized with β-actin levels on the same membrane.

### Transmission electron microscopy

Samples of soleus muscle were cut into ~1 mm^3^ fragments and fixed in 2.5% glutaraldehyde in 0.1M phosphate buffer, (pH 7.4) for 4 h at 4°C. After washing in phosphate buffer, tissues were post-fixed with 1% osmium tetroxide in phosphate buffer for 2 h at 4°C. Samples were dehydrated through graded alcohols (50, 70, 90, and 100%) and propylene oxide, and subsequently embedded in EMbed 812 resin (Electron Microscopy Science) (48 h at 60°C). Ultrathin (70 nm thick) sections were cut at a Sorvall Porter-Blum ultramicrotome, contrasted with uranyl acetate and lead citrate. The ultrastructure of the sample was visualized on a transmission electron microscope Philips 208S at Centro di Servizi Metrologici Avanzati (CeSMA, University of Naples Federico II); micrographs were acquired with a Mega View II Soft Imaging System camera. Mitochondrial compartment volume density was measured using the stereological point-sampling technique (Weibel et al., 1966; Altunkaynak et al., 2012). For each experimental group, 10 random images were taken at 25,000 x magnification. A grid with equally and symmetrically spaced intersection points was overlaid on each micrograph using the Image J1.50 software (NIMH). Mitochondrial volume density for each micrograph was calculated as the ratio of points overlapping the cellular compartment of interest and the total number of grid points overlapping the cytoplasm area. To measure mitochondrial fusion and fission percentage, the number of mitochondria undergoing fusion and fission on total mitochondrial number in the visual field was counted in 10 images for each experimental group.

### Statistical analysis

Data were analyzed by one-way ANOVA followed by the Bonferroni *post-hoc* test. Labeled means without a common letter differ (*P* < 0.05). Analyses were performed and plotted using GraphPad Prism (GraphPad Software, San Diego, CA, USA).

## Results

### Body weight and food intake

As expected, although different milk treatments provided similar total food intake, CM-treated animals exhibited a significant increase in body weight and body weight gain when compared with control, DM- and HM-treated rats (Table 1).

### Serum metabolites and inflammatory parameters

**Figure 1:**
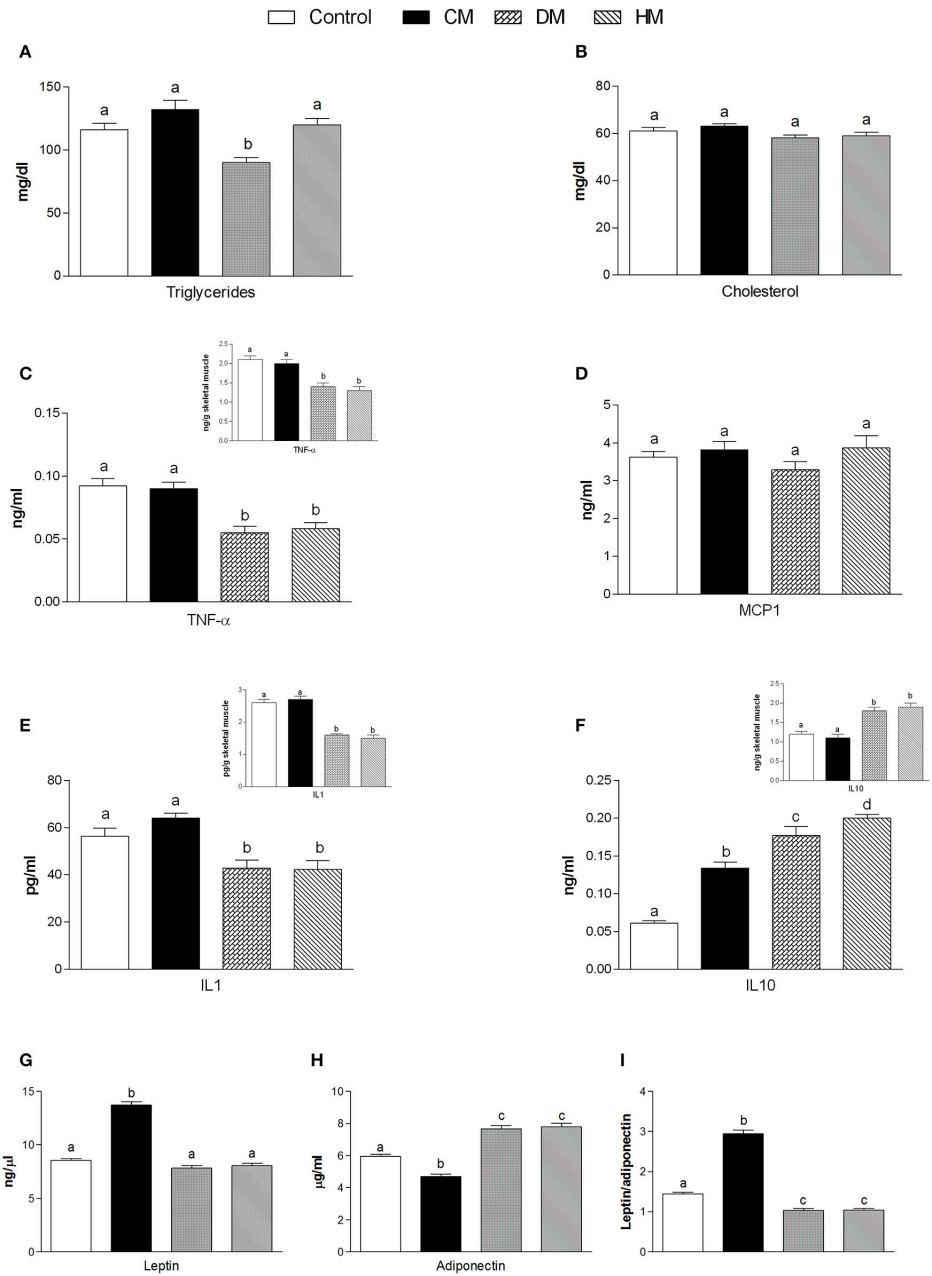
Effects of oral administration of human, donkey and cow milk on serum levels of lipids, hormones and inflammatory parameters. The graphs show serum levels of triglycerides **(A)**, cholesterol **(B)**, TNF-α **(C)**, MCP1 **(D)**, Interleukin- 1 (IL 1 **E**), interleukin-10 (IL 10 **F**), leptin **(G)**, adiponectin **(H)**, and leptin/adiponectin ratio **(I)**. Insets indicate skeletal muscle levels of TNF-α **(C)**, IL 1 **(E)**, and IL 10 **(F)**. Data are presented as means ± SEM from *n* = 7 animals/group. Labeled means without a common letter differ (*P* < 0.05).

**Figure 2:**
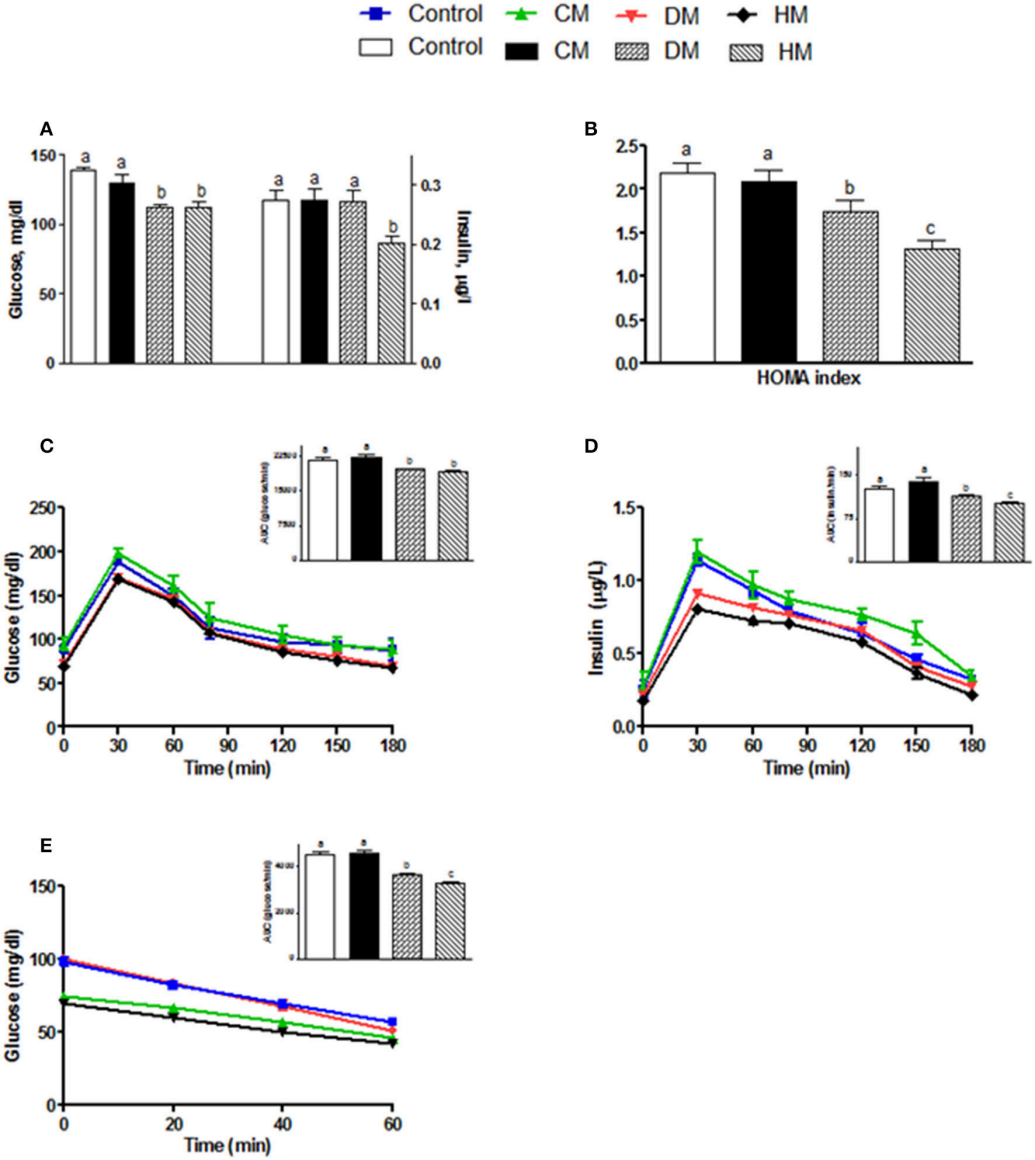
Effects of oral administration of human, donkey and cow milk on glucose homeostasis. The graphs show the plasma levels of glucose and insulin **(A)**, the Homa-index **(B)**, the plasma glucose **(C)**, and insulin **(D)** concentrations at different time points after glucose load, and insulin tolerance test **(E)**. The histograms in **(C–E)**, represent the area under curve (AUC) related to each condition. Data are presented as means ± SEM from *n* = 7 animals/group. Labeled means without a common letter differ (*P* < 0.05).

Serum parameters were reported in Figure 1. Triglycerides significantly decreased in the DM-fed animals (Figure 1A), while cholesterol and MCP1 serum levels were not affected by different milk treatments (Figures 1B,D, respectively). TNF-α and IL 1 levels significantly decreased in both serum and tissue of HM- and DM-fed animals in comparison with control and CM-fed rats (Figures 1C,E, respectively). IL 10 level increased in the serum of all treated groups (HM>DM>CM) compared to control (Figure 1F), while IL 10 level increased in the skeletal muscle of DM and HM compared to control and CM group (Figure 1F inset). Leptin concentration was significantly higher in CM-treated animals compared to the other groups; consistently, adiponectin concentration decreased in CM-fed animals, whereas it significantly increased in the DM- and HM-treated rat (Figures 1G,H). Accordingly, the leptin/adiponectin (L/A) ratio significantly increased in CM-treated rats compared to the other two groups (Figure 1I). Glucose levels were significantly lower in HM- and DM-fed rats compared to the other groups, while insulin levels were significantly lower only in HM rats (Figure 2A). Therefore, compared with controls, a marked reduction of HOMA index was observed in the DM and HM groups (HM<DM; Figure 2B). Moreover, HM- and DM-treated rats exhibited a higher tolerance to glucose load than CM-treated and control, (evaluated as “area under the curve” AUC of glucose levels during time); in agreement, the HM- and DM-fed animals showed a significant reduction of insulin release (AUC) after glucose load in comparison with the other groups (HM<DM) (Figures 2C,D). In addition, insulin tolerance test revealed better glucose reduction (AUC HM<DM), following insulin administration, in DM- and HM-fed rats compared with the CM-treated or control groups (Figure 2E).

### Skeletal muscle and liver lipid analysis

**Figure 3:**
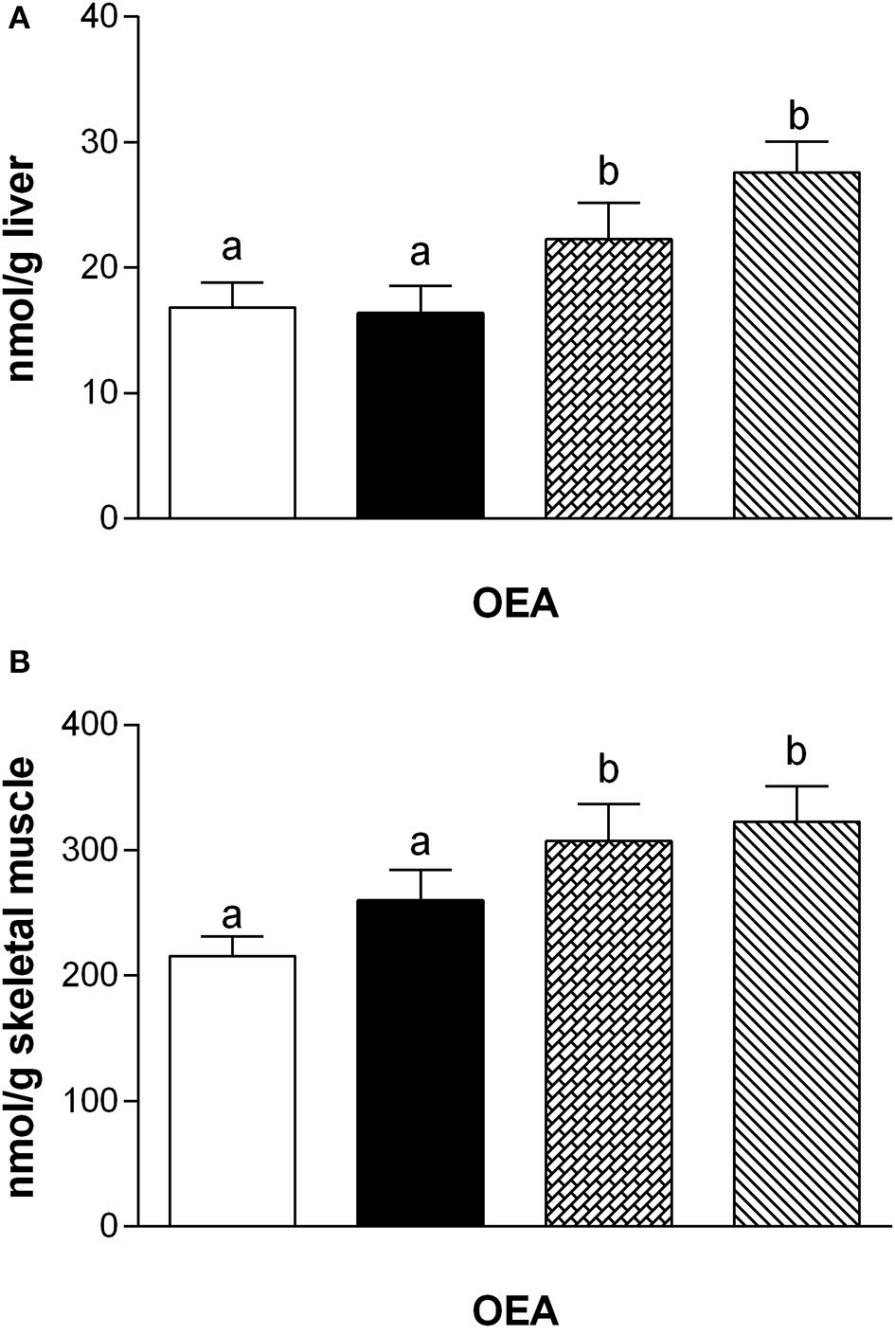
Tissue levels of oleylethanolamide (OEA). The graphs show the liver **(A)** and skeletal muscle **(B)** levels of OEA. Data are presented as means ± SEM from *n* = 7 animals/group. Labeled means without a common letter differ (*P* < 0.05).

Fatty acid analysis showed a significant increase of OEA in the liver and skeletal muscle of rats fed with HM and DM compared to CM or control animals (Figure 3), while the other amides and 2-AG did not change significantly, although PEA showed an increasing trend (Table 3).

**Table 3:** Tissue levels of AEA, PEA, and 2AG.

	**Control**	**CM**	**DM**	**HM**
**SKELETAL MUSCLE**				
AEA (nmol/g)	27.61 ± 2.36	18.03 ± 2.51	21.33 ± 2.07	22.99 ± 3.43
PEA (nmol/g)	106.79 ± 5.16	102.04 ± 15.64	115.91 ± 14.30	135.04 ± 12.29
2AG (nmol/g)	5979.3 ± 540.3	6646.9 ± 1016.3	7741.0 ± 730.0	7324.2 ± 480.1
**LIVER**				
AEA (nmol/g)	2.99 ± 0.89	3.97 ± 0.49	3.82 ± 1.16	3.14 ± 0.56
PEA (nmol/g)	33.23 ± 3.70	29.65 ± 5.19	38.52 ± 10.93	45.12 ± 7.06
2AG (nmol/g)	4112.1 ± 1169	4045.9 ± 588.2	5011.4 ± 1652.2	4608.0 ± 1461.3

*PEA, N-palmitoylethanolamine; AEA, anandamide; 2AG, 2-arachidonoyl glycerol. Values represent means ± SE from n = 7 animals/group*.

### Evaluation of mitochondrial parameters in skeletal muscle

**Figure 4:**
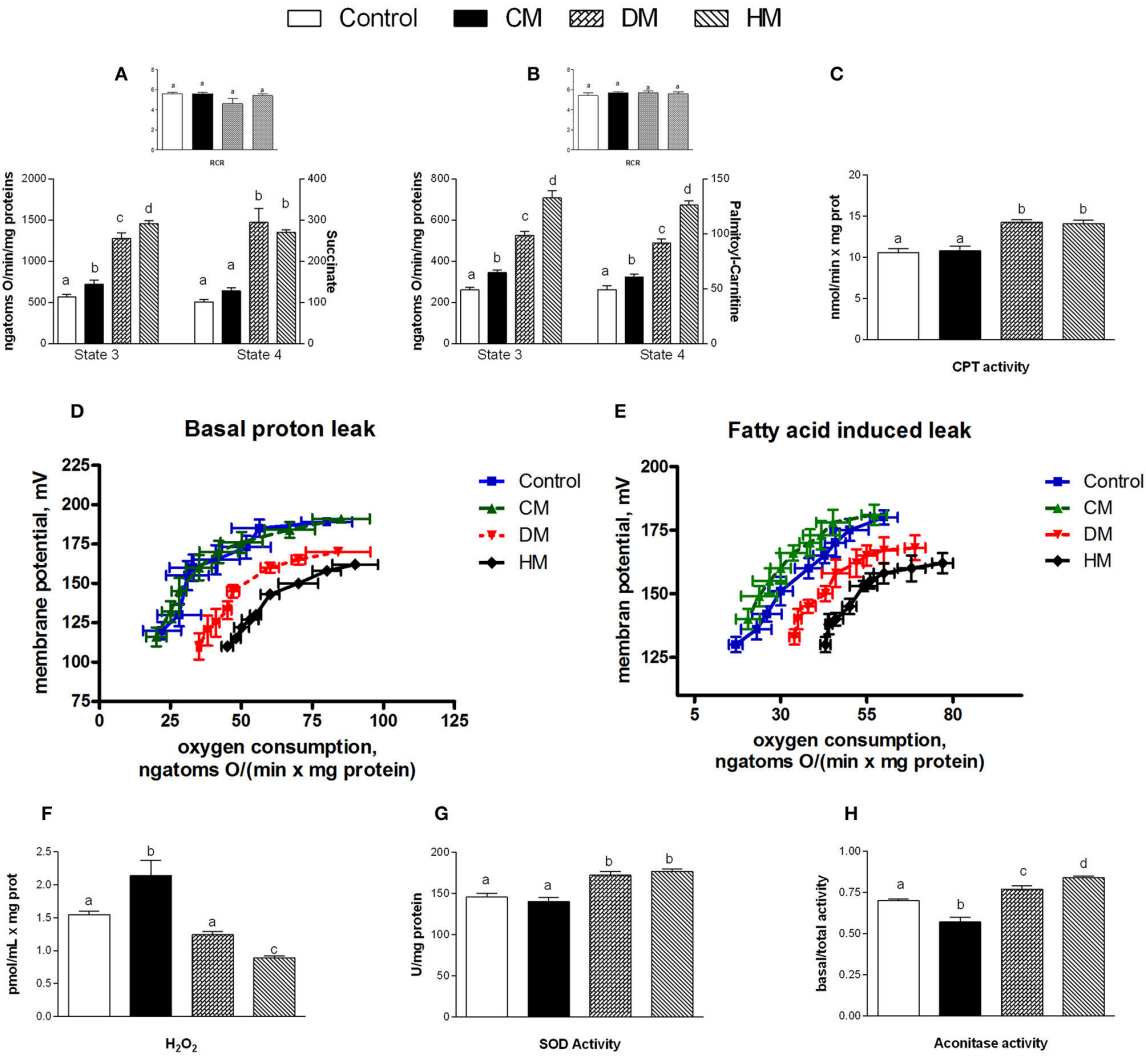
Effects of oral administration of human, donkey, and cow milk on energy efficiency, respiratory parameters, and oxidative stress in mitochondria isolated from skeletal muscle. The graph shows the skeletal muscle mitochondrial respiration rates measured in the presence of succinate **(A)** or palmitoyl-carnitine **(B)** as substrates, and the relative RCR (**A,B** inset), the carnitine-palmitoyl transferase (CPT) activity **(C)**, the basal **(D)**, and fatty acid-induced **(E)** proton leakage, H_2_O_2_ yield **(F)**, the superoxide dismutase (SOD) activity **(G)**, and the basal aconitase/total aconitase ratio **(H)**. Data are presented as means ± SEM from *n* = 7 animals/group. Labeled means without a common letter differ (*P* < 0.05).

When succinate was used as a substrate, state 4 respiration increased in mitochondria isolated from the DM- and HM- treated animals compared to the other groups (Figure 4A). In presence of palmitoyl-carnitine, state 4 increased in all three milk-treated groups, with the highest rate being observed in the HM animals (Figure 4B). Mitochondrial state 3 using both substrates, increased in the three milk-treated groups, with the highest oxidation rates observed again in the HM-treated group (Figures 4A,B). CPT activity increased in the DM- and HM-treated animals compared to control and CM-treated rats (Figure 4C). The RCR values did not significantly change among the groups, confirming the high-quality of mitochondrial preparations (Figures 4A,B insets). Mitochondria from control and CM-treated rats exhibited comparable kinetic curves of basal proton-leakage, that was significantly increased in DM-treated rats and even more in HM-treated ones (Figure 4D). Likewise, mitochondrial free fatty acid (FFA)-induced proton leakage was significantly increased in the DM- and HM-treated animals (HM>DM), but the effects on mitochondrial leakage in the CM-treated animals was the opposite, as it was slightly decreased compared to control (Figure 4E). Thus, the HM-treated animals exhibited the highest oxygen consumption to maintain the same membrane potential. H_2_O_2_ yield increased in CM-fed animals compared to controls, while it significantly decreased in DM- and at higher extent in HD-treated rats (Figure 4F). SOD activity was significantly higher in both DM- and HM-treated rats compared to control and CM-treated animals (Figure 4G). Similarly, aconitase activity was significantly increased in the DM-treated animals and, even more, in the HM-treated ones. In contrast, the CM-treated animals displayed a significant decrease in aconitase activity (Figure 4H). Altogether, these data suggest that the administration of DM and HM may result in diminishing the mitochondrial ROS emission.

### Antioxidant/detoxifying defenses

**Figure 5:**
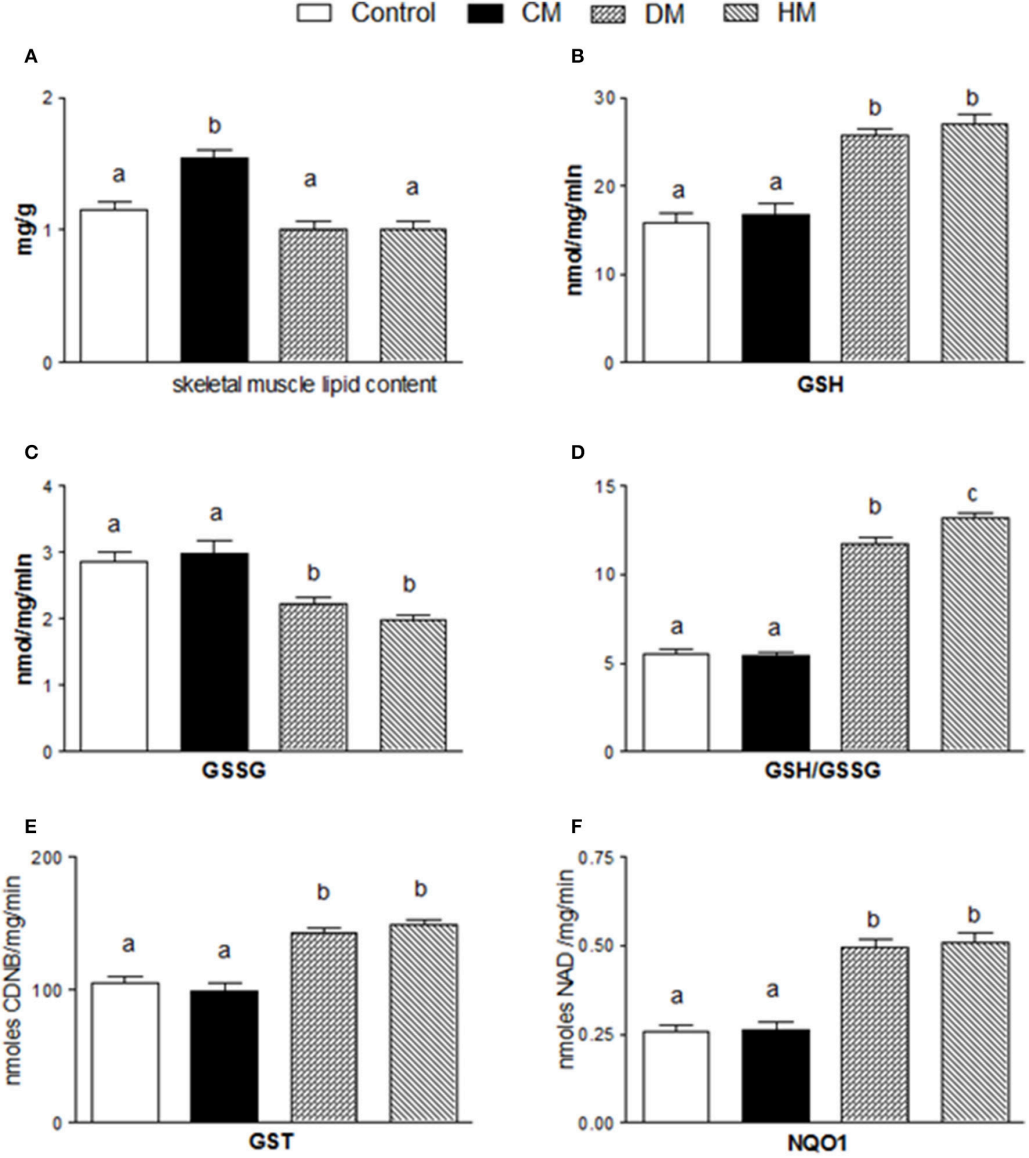
Effects of oral administration of human, donkey, and cow milk on lipid content and antioxidant/detoxifying defenses in skeletal muscle. The graphs show the total lipid content of skeletal muscle **(A)**, GSH content **(B)**, GSSG content **(C)**, the GSH/GSSG ratio **(D)**, the GST content **(E)**, and the NQO1 activity **(F)**. Data are presented as means ± SEM from *n* = 7 animals/group. Labeled means without a common letter differ (*P* < 0.05).

The lipid content in skeletal muscle of CM-treated rats was substantially higher than in the other groups (Figure 5A). Dietary supplementation with DM or HM improved the antioxidant state and cytoprotective enzyme activities. Indeed, while GSH levels significantly increased in DM or HM group, compared to the other groups (Figure 5B), GSSG content was reduced, resulting in an increased GSH/GSSG ratio in the HM and DM groups (HM>DM) (Figures 5C,D). In addition, the activities of GST and NQO1 were significantly higher in the DM- or HM-treated rats than in the CM-treated and control rats (Figures 5E,F).

### Mitochondrial dynamics

**Figure 6:**
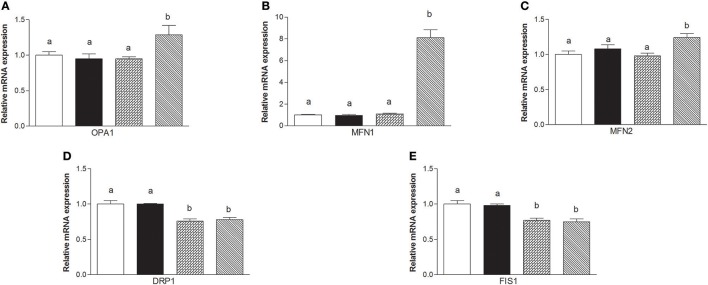
Effects of oral administration of human, donkey, and cow milk on skeletal muscle mitochondrial dynamics. The graphs summarizing the results of real time PCR represent the relative levels of mRNAs for OPA1 **(A)**, MFN1 **(B)**, MNF2 **(C)**, DRP1 **(D)**, and FIS 1 **(E)**. Data are presented as means ± SEM from *n* = 7 animals/group. Labeled means without a common letter differ (*P* < 0.05).

**Figure 7:**
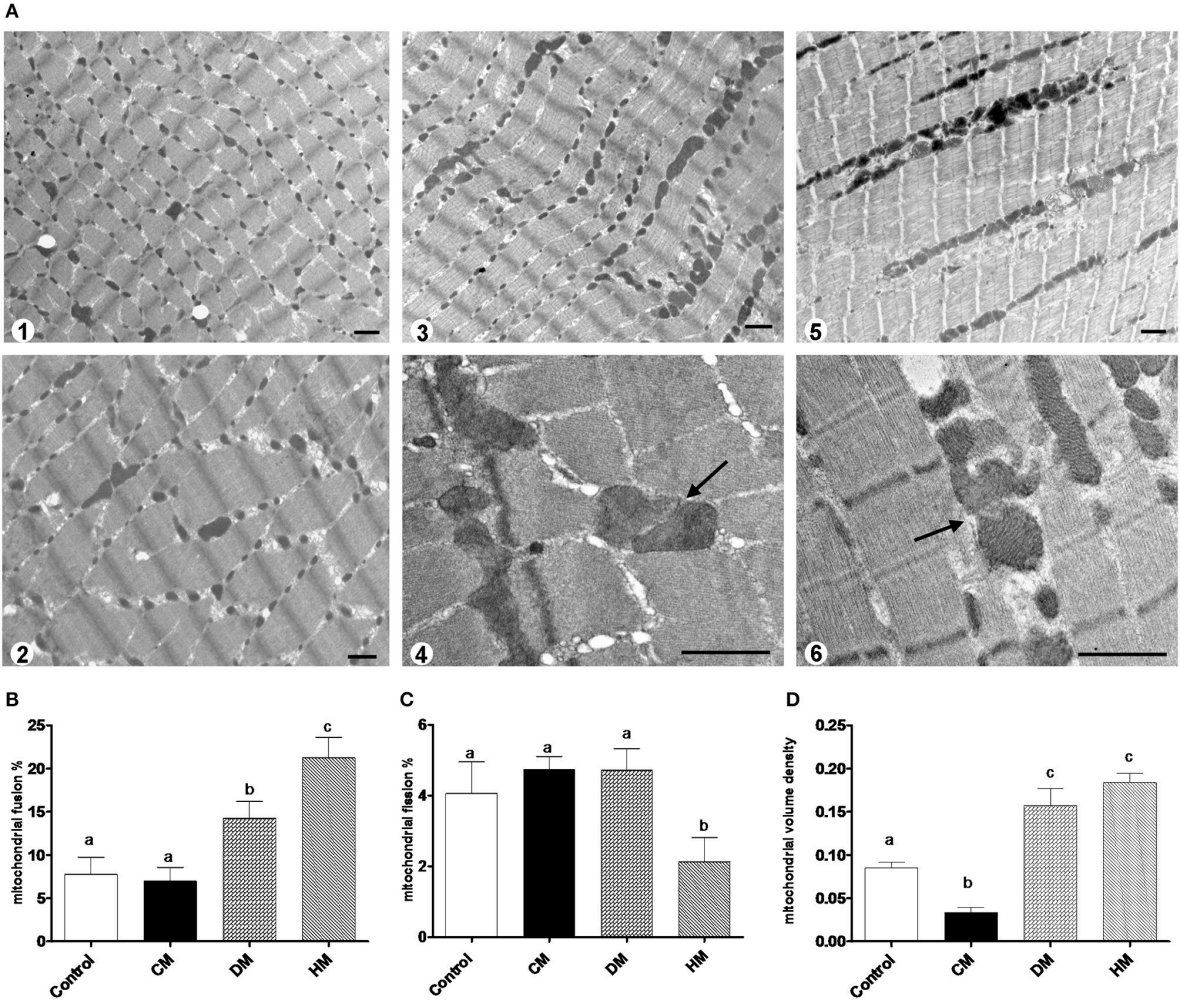
Mitochondrial morphology and dynamics. The photomicrographs are the transmission electron microscopic images of the soleus muscles taken from the control **(A1)**, CM **(A2)**, DM **(A3,4)**, and HM-treated animals **(A5,6)**. Mitochondrial fusion was observed more frequently in the samples of DM **(A3,4)** and HM animals **(A5,6)**. Arrows indicate the fusion points. Scale bars, 1 μm. Mitochondrial fusion and fission are quantified as percentage of mitochondria undergoing fusion **(B)** and fission **(C)** in reference to the total number of the mitochondria in the visual field. **(D)** Mitochondrial volume density. Data are presented as means ± SEM from *n* = 7 animals/group. Labeled means without a common letter differ (*P* < 0.05).

**Figure 8:**
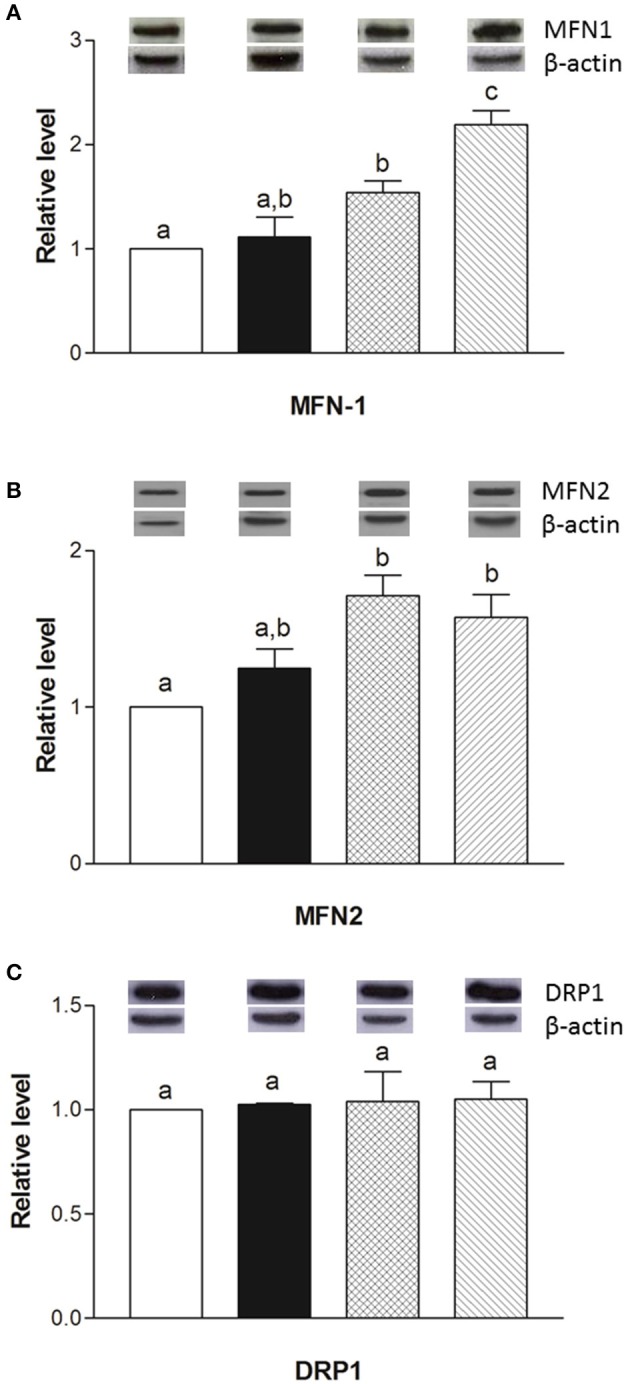
Effects of oral administration of human, cow, and donkey milk on the protein levels of MFN1, MFN2, and DRP1. Results of the quantitative Western blot analyses for MFN1 **(A)**, MFN2 **(B)**, and DRP1 **(C)** were summarized. Protein levels of MFN1, MFN2, and DRP1 assessed in densitometry were normalized to the level of β-actin. Insets: representative immunoreactive signals for MFN1, MFN2, DRP1, and β-actin. Data are presented as means ± SEM from *n* = 7 animals/group. Labeled means without a common letter differ (*P* < 0.05).

The mRNA levels of *Opa1, Mfn1*, and *Mfn2* significantly increased in HM-fed rats compared to other groups (Figures 6A–C). On the other hand, *Drp1* and *Fis1* mRNA levels in DM and HM significantly decreased when compared to CM or control groups (Figures 6D,E). These results were consistent with the morphological data on the ultrastructure of the skeletal muscles, in fact, mitochondria from DM and HM groups appeared more abundant and larger than those in the control group (Figure 7A). This was confirmed by a higher fusion percentage in HM and DM (Figure 7B) and a lower percentage of fission in HM (Figure 7C), as well as an increased mitochondrial volume density in DM and HM (Figure 7D). Moreover, the mitochondria of DM and HM appeared more electron-dense compared to those in CM and control groups. At the molecular level, the expression of two marker proteins for mitochondrial fusion, MFN1 and MFN2, was evidently increased in the DM and HM in comparison with control and CM animals (Figures 8A,B), while the expression levels of the marker protein for fission, DRP1, did not significantly change among all groups (Figure 8C).

## Discussion

The main finding of this study is that oral supplementation with HM and DM of rat diet affects glucose and lipid metabolism, modulating pro- and anti-inflammatory serum and tissue mediators. In particular, HM and DM exert their beneficial effects on skeletal muscles by reducing fat accumulation and modulating mitochondrial function, efficiency, and dynamics associated to an improvement of the redox status and activation of detoxifying enzymes (Nrf2 pathway). Our study highlights the ability of HM, and to a lesser extent of DM, to control glucose homeostasis, improving HOMA index and glycaemic and insulin response, as demonstrated by the responses to glucose load and insulin tolerance tests. These results are consistent with other data indicating that HM, compared with CM, lowers insulin blood levels (Gunnerud et al., 2012). It has been shown that insulin response to glucose can be modulated not only by the lactose component, but also to the whey fraction, or the bioactive peptides that are present in the milk (Nilsson et al., 2004, 2007). Here, we have shown that the modulation of glucose metabolism by HM and DM, strongly correlates with the reduction of inflammatory mediators in serum and tissue. Moreover, we have shown that the modulation of glucose metabolism may be related to adiponectin and leptin balance. These two hormones, derived from fat cells, are involved not only in glucose and lipid metabolism, controlling energy homeostasis, but also in the modulation of inflammation (Minokoshi et al., 2002; Yamauchi et al., 2002). With the accumulation of fat mass, leptin level increases, while adiponectin decreases. Accordingly, CM-treated group, characterized by higher body lipid levels compared with the HM and DM groups (Trinchese et al., 2015), showed increased leptin level and decreased adiponectin level. Interestingly, HM and DM-treated animals, with a body lipid level similar to control (Trinchese et al., 2015), displayed significantly increased adiponectin in their sera compared with other groups. In some animal models, a decrease in adiponectin level was found to parallel to a reduction in insulin sensitivity and to precede the onset of type 2 diabetes (Chakraborti, 2015). Adiponectin secretion is inhibited by several factors including high level of TNF-α and oxidative stress (Chakraborti, 2015). Hence, our data, showing increased adiponectin level in HM and DM groups, associated with decreased level of TNF-α, may indicate a lower grade of inflammation in these animals. It has been proposed that a useful index of metabolic diseases is the ratio of leptin to adiponectin (L/A), which shows a better correlation to insulin resistance than the level of leptin and adiponectin alone (Oda et al., 2008). Our data indicates a significantly lower L/A ratio in DM and HM groups, that correlates with the modulation of glucose metabolism in these animals. Adiponectin and leptin have been recently detected in HM (Lönnerdal, 2013), and it has been postulated that these adipokines may be involved in the regulation of growth in infancy and influence the programming of energy balance with long-term consequences on health (Guardamagna et al., 2012; Agostoni et al., 2013). Indeed, it has been demonstrated that breastfeeding is associated with a reduced risk of type 2 diabetes, with lower blood glucose and serum insulin concentrations during infancy and with lower insulin levels in adulthood (Owen et al., 2006). Our study suggests that DM affects glucose metabolism in a similar way HM does, although the biologically active components of DM remain largely unknown.

Interestingly, HM and DM have high concentration of palmitic acid in the sn-2 position of triacylglycerol backbone (Innis, 2016), that can be responsible for the increased levels of OEA that we found in liver and skeletal muscle of HM and DM animals. These increased levels of OEA may have important implications in terms of energy metabolism and inflammation. Indeed, OEA, recently identified as an important regulator of inflammation, is an endogenous ligand of the nuclear receptor PPAR alpha, which regulates fatty acid metabolism in different tissue. In particular, PPARα activation increases peroxisomal and mitochondrial oxidation and leads to anti-inflammatory and antioxidant effects (Contreras et al., 2013; Pontis et al., 2016).

**Figure 9:**
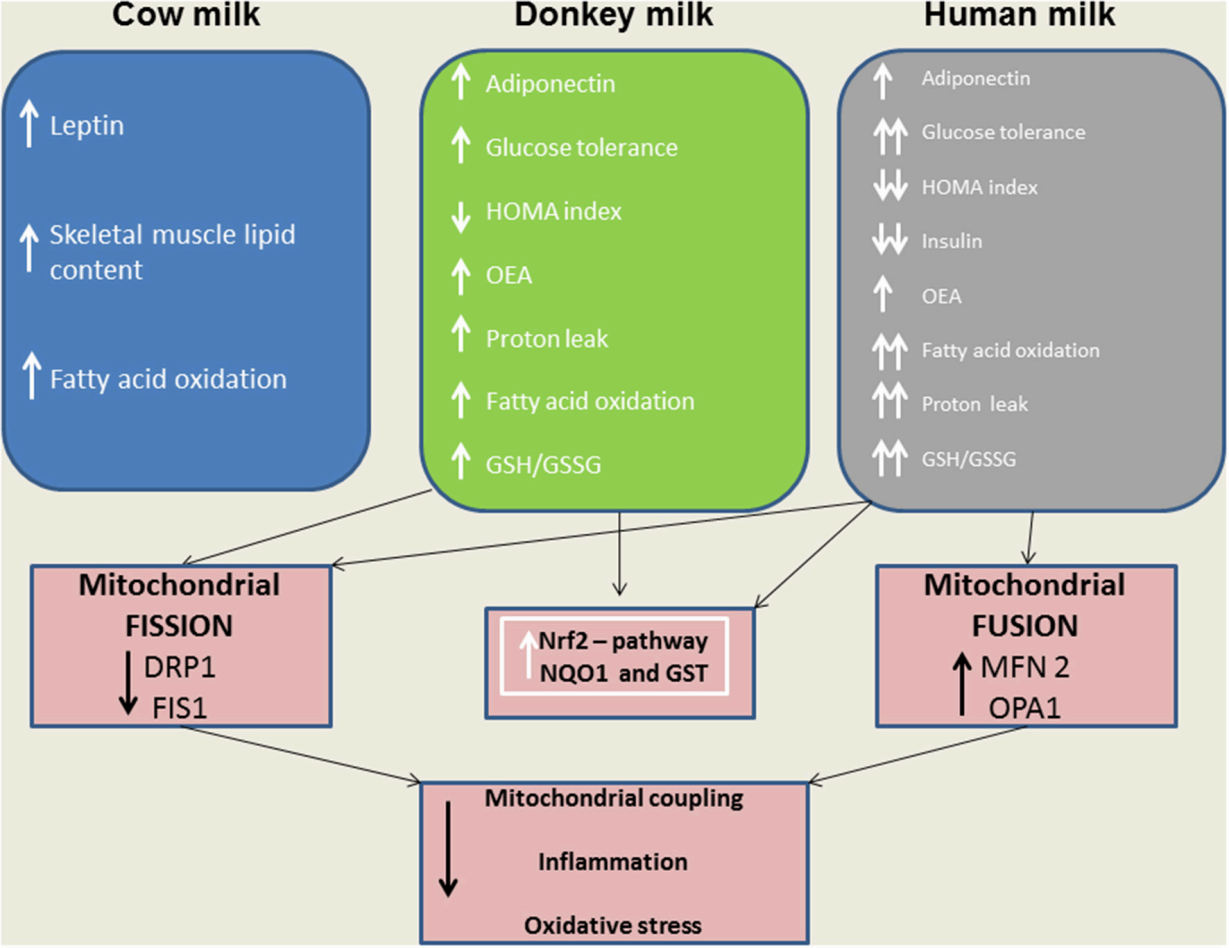
A model depicting the effects of distinct milk supplementation. The beneficial effects elicited by HM and DM are, at least in part, mediated by their ability to diminish mitochondrial ROS emission, and to modulate mitochondrial uncoupling and dynamics as well as Nrf2 activation.

Considering the central role of the skeletal muscle in lipid and glucose metabolism, we evaluated the effects of different milks on skeletal muscle mitochondria in terms of their metabolic function, efficiency and dynamics. We observed that DM, and even more HM, had several beneficial effects. For instance, both HM- and DM skeletal muscle mitochondria showed increased respiratory capacity and improved redox status even when the ability to utilize fat as a metabolic fuel was increased. The increased mitochondrial fatty acid oxidation observed in the skeletal muscle is likely to be related to an enhancement of CPT activity, which would further increase entry of long-chain FFAs into mitochondria. The consequent increase in lipid oxidation is apparently sufficient to handle the decreased load of skeletal muscle FFAs. In addition to stimulating fatty acid oxidation, HM induced a less efficient utilization of lipid substrates through the stimulation of thermogenic mechanisms such as proton-leakage. This decline in mitochondrial energy efficiency may also contribute to fat burning. Therefore, we hypothesized that HM-fed animals, similarly to DM-treated rats, might be protected from development of obesity and insulin resistance through these mechanisms. The increase in proton-leakage has a further metabolic implication that is the maintenance of mitochondrial membrane potential below the critical threshold for ROS production (Skulachev, 1991; Mailloux and Harper, 2011). Accordingly, in HM- and DM-treated animals, we observed a decrease in H_2_O_2_ yield, an enhancement of antioxidant defense mechanisms (aconitase inhibition, SOD activity), and an improvement of redox status (GSH/GSSG ratio), as well as increased activities of detoxifying enzymes (GST-NQO1) which are at least in part attributable to the activation of the Nrf2–ARE pathway. Another important observation in our study is that DM and HM improve glucose metabolism modulating mitochondrial dynamics. Mitochondrial dynamic is a complex process that impacts on mitochondrial metabolism through complex modulation of proteins that play a key role in fusion and fission machinery. The fusion protein Mfn2, a potent modulator of mitochondrial metabolism, also controls cell metabolism and insulin signaling by limiting reactive oxygen species production, and is subjected to tight regulation. Indeed, proinflammatory cytokines, glucocorticoids, or lipid availability block its expression, whereas exercise and increased energy expenditure promote its upregulation (Zorzano et al., 2015; Schrepfer and Scorrano, 2016). Our results show that HM and DM decrease Drp1 and Fis1 mRNA levels, while HM increases the levels of mRNA coding for mitochondrial fusion proteins (OPA1, Mfn1, and Mfn2), suggesting a shift toward fusion in these animals. The increase in mitochondrial fusion in the skeletal muscles of HM and DM animals was confirmed by western blot and electron microscopy analysis. This latter analysis also revealed that mitochondria of DM and HM are more abundant, larger and more electron-dense than those in the CM and control animals. The higher electron-density of DM and HM could be linked to mitochondrial state, in particular to the inner membrane condensation (Mannella, 2006; Sancho et al., 2014). Condensed state (alternative to orthodox state) has been associated to more active mitochondria, suggesting that these organelles regulate their shape to adjust their activity with metabolic conditions (Mannella, 2006). Based on these data, we propose that the shift toward fusion, upon DM and HM administration, contribute to the improvement of glucose metabolism in these animals. Accordingly, previous data indicated that mitochondrial fragmentation and enhancements of fission machinery negatively affected glucose metabolism, leading to an increase in ROS formation due to alterations in mitochondrial electron transport and coupling (Yu et al., 2008; Westermann, 2012). Our data indicate that both HM and DM have a protective role on metabolism, with HM having higher beneficial effects. These potentiated effects of HM may depend on molecular mechanisms involving modulation of fusion/fission processes and consequently diminished levels of inflammation, ROS, and improved glucose metabolism. A shift toward fission results in numerous mitochondrial fragments contributing to the elimination of irreversibly damaged mitochondria through autophagy. At the same time, the mixing of the matrix and the inner membrane allows the respiratory machinery components to cooperate most efficiently, reducing the uncoupling effect. On the other hand, a shift toward fusion optimizes mitochondrial function and thereby plays a beneficial role in maintaining long-term bioenergetics capacities. Indeed, fusion favors generation of interconnected mitochondria, which may be responsible for a decrease in mitochondrial efficiency stimulating the uncoupling effect that contributes to the dissipation and rapid provision of energy. Based on these findings, we can identify the activation of mitochondrial fusion and the uncoupled respiration as part of a complex protective mechanism that reduces oxidative stress and maintains healthy and functional mitochondria (Li et al., 2000; Liesa et al., 2009; Liesa and Shirihai, 2013). In agreement with this hypothesis, Mfn2-depleted cells exhibited a decreased mitochondrial proton leak and increased bioenergetics efficiency (Bach et al., 2003), suggesting that the loss-of-function of Mfn2 in obesity condition may enhance bioenergetics efficiency and thereby contribute to obesity by reducing energy expenditure and increasing fat energy store (Liesa et al., 2009). In conclusion, our study highlights that dietary supplementation with HM or DM decreases inflammatory factors, modulates lipid and glucose metabolism, and increases OEA tissue levels (Figure 9). Interestingly, we indicate that the beneficial effects elicited by HM and DM are, at least in part, mediated by their ability to improve redox status, modulating Nrf2 activation, mitochondrial uncoupling, and dynamics.

## Ethics statement

The procedures reported here, involving animals and their care, were approved by the Institutional Committee on the Ethics of Animal Experiments (CSV) of the University of Naples Federico II and by the Ministero della Salute.

## Author contributions

MPM, LG, and MC: Conceived the original idea, designed, and supervised the whole study; GT, GC, CDF, SA, MP, EP, LM, and AD: Performed the experiments; MPM and MC: analyzed and interpreted data; MPM, JTC, RN, SB, AC, GMR, RM, RBC, LG, and MC: Critically revised the manuscript for intellectual content; MPM and MC: wrote the paper.

### Conflict of interest statement

The authors declare that the research was conducted in the absence of any commercial or financial relationships that could be construed as a potential conflict of interest.
